# Survey on Blockchain-Based Data Storage Security for Android Mobile Applications

**DOI:** 10.3390/s23218749

**Published:** 2023-10-26

**Authors:** Hussam Saeed Musa, Moez Krichen, Adem Alpaslan Altun, Meryem Ammi

**Affiliations:** 1 Faculty of Technology, Department of Computer Engineering, Selçuk University, 42130 Konya, Turkey; hs.musa@alaqsa.edu.ps (H.S.M.); altun@selcuk.edu.tr (A.A.A.); 2 Faculty of Computer Science and Information Technology, Al-Baha University, Al Baha 65431, Saudi Arabia; 3 ReDCAD Laboratory, National School of Engineers of Sfax, University of Sfax, Sfax 3000, Tunisia; 4 Digital Forensics Department, Criminal Justice College, Naif Arab University for Security Sciences, Riyadh 14812, Saudi Arabia; mammi@nauss.edu.sa

**Keywords:** mobile applications, blockchain, data storage, android, survey, current practices, challenges, future directions, blockchain based Secure Android Data Storage (BSADS)

## Abstract

This research paper investigates the integration of blockchain technology to enhance the security of Android mobile app data storage. Blockchain holds the potential to significantly improve data security and reliability, yet faces notable challenges such as scalability, performance, cost, and complexity. In this study, we begin by providing a thorough review of prior research and identifying critical research gaps in the field. Android’s dominant position in the mobile market justifies our focus on this platform. Additionally, we delve into the historical evolution of blockchain and its relevance to modern mobile app security in a dedicated section. Our examination of encryption techniques and the effectiveness of blockchain in securing mobile app data storage yields important insights. We discuss the advantages of blockchain over traditional encryption methods and their practical implications. The central contribution of this paper is the Blockchain-based Secure Android Data Storage (BSADS) framework, now consisting of six comprehensive layers. We address challenges related to data storage costs, scalability, performance, and mobile-specific constraints, proposing technical optimization strategies to overcome these obstacles effectively. To maintain transparency and provide a holistic perspective, we acknowledge the limitations of our study. Furthermore, we outline future directions, stressing the importance of leveraging lightweight nodes, tackling scalability issues, integrating emerging technologies, and enhancing user experiences while adhering to regulatory requirements.

## 1. Introduction

### 1.1. General Context

The mobile applications (apps) industry is constantly evolving, with new advancements and trends appearing on a regular basis [1,2,3]. There has been a recent trend toward developing more advanced secure apps that incorporate artificial intelligence [4,5,6,7]. Furthermore, businesses have increasingly turned to mobile apps for e-commerce, offering their products and services through exclusive, secure mobile platforms. The widespread use of smartphones has greatly aided the expansion of the mobile app market [8,9]. These devices, which combine the features of a computer with the portability of a cell phone, have become an essential part of many people’s daily lives. As a result, there has been an increase in demand for secure apps that can be used on these devices [10].

The presence of malicious apps designed to steal sensitive information or spread malware is a major issue [11]. Another issue is the possibility of insecure data storage and transmission, which exposes sensitive information to malicious individuals [12,13]. Addressing these security concerns in the mobile app market is critical, as mobile device and app usage is expected to rise in the coming years [14,15]. Neglecting these security concerns can have serious consequences, including the loss of sensitive information and financial harm to both individuals and organizations, potentially resulting in data breaches, identity theft, and financial losses [16,17]. Furthermore, there are concerns about data privacy because many apps collect and share personal information without the user’s knowledge or consent [18].

In blockchain-based systems, the data is stored in distributed ledgers, verified through network nodes by consensus algorithms constituting a decentralized system and affording users’ trust. These systems store data in an encrypted and immutable manner to provide security. They manage the data across all of the nodes in a peer-to-peer network, so there is no possibility for a single point of failure which ensures integrity in the network. They process data easily, saving time and giving control to the user rather than to a centralized authority, providing simplicity; finally, the use of encryption and digital signatures via blockchain technology provides the user with privacy [19,20,21,22,23,24,25,26]. It is expected that blockchain technology will be the future of mobile apps.

### 1.2. Motivation and Main Contributions

The use of mobile apps to store sensitive information such as personal identification documents, financial information, and medical records has grown in popularity, making data storage security in these apps a critical concern. Data storage security entails safeguarding data against unauthorized use, disclosure, modification, disruption, or destruction. As a result, it is critical to ensure that this information is securely stored and protected from unauthorized access. The purpose of this paper is to identify any challenges or limitations associated with the use of blockchain technology in the security of Android mobile app data storage. The focus on Android is justified by its status as the dominant mobile operating system worldwide, commanding nearly 70% of the market share [27]. Furthermore, statistical reports from Statista indicate that Android is also the most vulnerable operating system [28].

Our main contributions can be summarized as follows:

1. Providing an overview of the state-of-the-art in blockchain technology integration with mobile app data storage security.
2. Identifying the limitations and challenges of using blockchain technology for mobile app data storage security.
3. Identifying the future directions of blockchain technology.
4. Suggesting optimization strategies to overcome the limitations discussed.
5. Proposing a Blockchain-based Secure Android Data Storage (BSADS) framework.

### 1.3. Security Issues and Measures

Mobile threats are classified as web-based, app-based, network-based, or physical in nature. Device loss or theft, mobile malware, spyware, untrusted applications, storing sensitive information without proper encryption, poor authentication and authorization, unwanted calls and SMS, phishing, rooting, privacy breaches, vulnerable development, and weak default configurations are examples of these threats [29]. These threats pose risks such as data loss, identity theft, financial fraud, unauthorized access to sensitive information, and device and data damage. With so many challenges, it is necessary to implement strong security measures to protect mobile devices and their data. Kaspersky discovered nearly 3.5 million malware apps on over 1 million devices in 2014. By 2017, Kaspersky’s in-lab detection technologies were processing 360,000 malicious files per day, with malware programs accounting for 78% of those files. This means that over 280,000 malware files were detected every day, with many of them aimed at mobile devices. According to Kaspersky, the following are the top seven mobile device threats: unsecured Wi-Fi, data leakage, network spoofing, spyware, phishing attacks, broken cryptography, and improper session handling [30].

The Open Web Application Security Project (OWASP) also gathered information about security vulnerabilities and published a list of the top ten risks for mobile devices [31]. The threats are categorized as follows: improper platform usage, insecure communication, insecure authentication, lack of cryptography, insecure authorization, poor client code quality, code manipulation, reverse engineering and insecure data storage [32,33,34]. Real-life examples of mobile app security breaches serve as cautionary tales for developers and users alike. One instance involved a security flaw in Amazon’s Ring Neighbors app, which exposed the precise locations and home addresses of users who made public posts, even though the posts were originally meant to be public but not location-specific. Another incident occurred with the Slack mobile app, which inadvertently logged user credentials in clear text on devices, prompting affected users to change their passwords. SHAREit, a popular Android file-sharing app with over 1 billion downloads, faced vulnerabilities that allowed for the execution of malicious code on smartphones for an extended period before being patched. Furthermore, 13 Android apps utilizing insecure third-party cloud services put the personal information of up to 100 million users at risk. ParkMobile experienced a breach affecting up to 21 million users, attributed to the improper handling of third-party software, leading to a class-action lawsuit. Klarna’s mobile banking app also suffered a security breach due to human error, temporarily exposing account information to other users. Lastly, hackers exploited the design flaws of the Portpass Canadian COVID vaccination passport app, compromising the privacy of 650,000 users due to unencrypted personal data [35]. These incidents underscore the need for mobile app developers to prioritize security, undergo comprehensive training, and address data storage challenges with utmost seriousness.

Various security measures can be implemented by developers, users, and app hosting providers to safeguard mobile devices from security threats [33]. Developers play a critical role in ensuring app security by adhering to secure coding practices, including the use of robust cryptographic algorithms with long keys and TLS/SSL for secure communication. Regular issuance of security updates and the inclusion of security patches in their libraries are recommended practices. Users also have responsibilities in protecting their devices and data, such as keeping their operating systems and apps up to date, refraining from device rooting, and being cautious when installing unfamiliar apps. It is advisable for users to download apps exclusively from reputable sources like the Google Play Store, which conducts checks for malicious code. Downloading apps from third-party or internet sources can pose security risks.

Hosting providers, like the Google Play Store, serve as trustworthy platforms for Android apps and actively monitor and disable apps with security vulnerabilities. Using techniques such as static and partial dynamic analysis, this app store can assign security scores to mobile apps based on their security measures, potentially influencing developers to implement robust security practices to improve their apps’ visibility in searches and recommendations. This approach empowers users to make informed decisions about app installations based on their security scores. While millions of apps are available on the widely used Google Play Store, the exponential growth of mobile app usage has raised concerns about security. Vulnerabilities that expose sensitive information have been identified in many apps, and even official app stores have hosted malicious apps. To address these issues, mobile app developers and device manufacturers should consider various security measures, including the adoption of blockchain technology, as suggested by our study [36].

Many suggested solutions and recommendations have been made by researchers to enhance the security of data storage in Android OS. Physical and software threat solutions have been proposed, like using Clean OS, TinMan, Sentry, Armored, Deadbolt, and DroidVault for physical threats and Android Encryption for software threats [37]. Still, more research is required as new technologies like blockchain are being developed to address the challenges facing data storage security and provide more secure, high-performance, scalable, cost-effective, and reliable data storage solutions.

### 1.4. Structure of the Article

This paper is structured into six main sections, it begins with an Introduction section where we provide an overview of the mobile app industry’s security challenges and the motivation for our study. In the Blockchain Technology and Data Storage section, we explore the fundamentals of blockchain technology, its components, types of networks, and data storage mechanisms. Moving on to the Related Work section, we conduct a literature review and history of blockchain in mobile apps and identify research gaps. The Blockchain-Based Mobile Apps Framework section introduces the need for blockchain in Android data storage, outlines the advantages of blockchain over traditional encryption methods, and presents our proposed framework—the Blockchain-based Secure Android Data Storage (BSADS) framework—with detailed discussions of its six layers. It also addresses various obstacles in adopting blockchain for mobile apps, from data storage costs to scalability and mobile-specific constraints with proposed solutions to these challenges, including blockchain pruning, off-chain solutions, and energy-efficient consensus algorithms. The Future Directions and Recommendations section explores potential paths forward, such as leveraging lightweight nodes and integrating with emerging technologies. Finally, we conclude with a recap of our key findings in the Conclusions section, emphasizing the promise of blockchain in enhancing Android mobile app data storage security, while noting that ongoing research holds the key to unlocking its full potential.

## 2. Blockchain Technology and Data Storage

**Figure 1:**
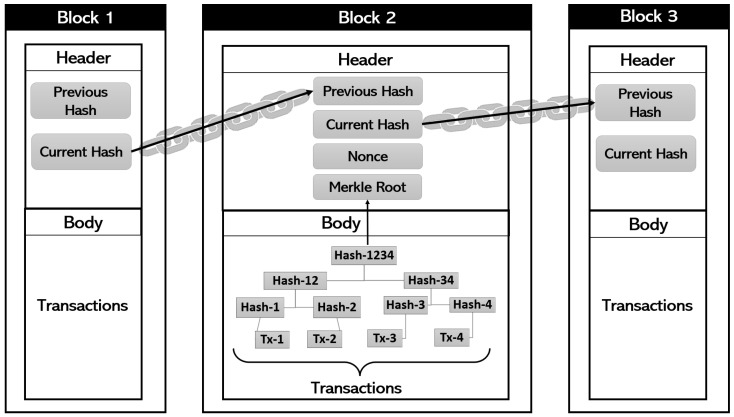
Blockchain general architecture.

Blockchain technology was introduced by Satoshi Nakamoto in 2008 [38,39,40,41]. Blockchains are decentralized digital record-keeping systems that consist of transactions that are verified using cryptography and grouped together into blocks [42,43,44,45]. Together, the blocks are linked in a secure way, making it easy to detect any tampering [46]. As more blocks are added, it becomes harder to make changes to older blocks, making the chain of blocks tamper-resistant. The ledger is duplicated across the network, and any discrepancies are automatically resolved based on established protocols [47]. The general architecture of a blockchain is shown in Figure 1.

Away from blockchain technology, various approaches to data storage, including relational databases like MySQL or Oracle, NoSQL databases such as MongoDB or Cassandra, centralized storage solutions, and cloud-based storage solutions all offer a range of advantages and drawbacks. Relational databases provide strong consistency and transactional support but can be complex to manage and may not scale well for extensive datasets. NoSQL databases prioritize flexibility and scalability but may sacrifice some consistency for performance and can be challenging to query and maintain. Centralized storage offers simplicity and high performance but is vulnerable to security risks and lacks transparency. Cloud storage, while cost-effective and convenient, raises concerns about data privacy and security due to offsite storage. In general, the choice of data storage approach will depend on the specific needs and requirements, as well as the available resources and expertise [48,49,50,51,52,53]. As we are concentrating on the use of blockchain for data storage security, let us explore that thoroughly.

### 2.1. Blockchain Components

In this subsection, we delve into the core components of the blockchain system to gain a better understanding of the technology [54,55]. Transactions form the foundation of a blockchain, as data is organized into blocks and arranged in a specific order [56]. In decentralized blockchain networks, transactions are initiated using private key cryptography, initially stored in an unconfirmed transaction pool. These transactions undergo validation by peers, and upon confirmation by miners and verification through the network’s consensus mechanism, they are added to a block and subsequently included in the immutable ledger maintained by each peer. Mining, a pivotal process, involves the validation and addition of transaction records to blocks using linked-list-based data structures. Miners undertake this task by solving computational puzzles through consensus algorithms like Proof of Work (PoW). While this process continually expands the blockchain, valid solutions can sometimes lead to delays or forks in the blockchain, with miners actively monitoring the network to choose which fork to mine on. Blockchain maintenance, therefore, becomes a collaborative and decentralized effort among miners [57]. Achieving consensus poses a unique challenge in a trustless environment with no centralized authority. Various consensus protocols have been developed to address this challenge and ensure agreement among nodes on the blockchain’s current state and the validity of new transactions. These protocols draw parallels to the Byzantine generals problem, where generals must agree despite potential traitors in their midst. Multiple consensus algorithms are employed in blockchain systems, including Proof of Work (PoW), Practical Byzantine Fault Tolerance (PBFT), Tendermint, Proof of Stake (PoS), and Delegated Proof of Stake (DPoS), each with distinct characteristics and considerations. Finally, while not present in all blockchains, smart contracts play a pivotal role in networks like Ethereum. Smart contracts are self-executing agreements with contractual terms expressed in code [58,59]. They can be stored on blockchain networks and facilitate asset exchanges without intermediaries [60,61,62,63,64,65,66]. Furthermore, smart contracts can serve as a means of data storage, encoding data directly into the contract to create a tamper-proof and permanent record accessible and verifiable by authorized parties using cryptographic keys. Smart contracts [67] offers a secure and transparent approach to decentralized data storage in mobile apps. However, they are not without limitations, such as deployment costs, execution complexity, and potential code vulnerabilities.

### 2.2. Types of Blockchain Networks

The type of blockchain network can be determined by its permission model, which decides who has the ability to maintain it (e.g., adding blocks).

Table 1 provides an overview of the three main types of blockchain networks: permissionless (public or open), permissioned (private or closed), and hybrid [19,20,21,22,24,68,69,70,71,72]. For each type, the table summarizes the details, benefits, usage, and main challenges. Permissionless blockchain networks are decentralized, transparent, and validated by a network of nodes through a consensus mechanism. They are suitable for decentralized apps, such as Bitcoin, and apps that require transparency, security, and decentralization, such as smart contracts and supply chain management. However, they face challenges such as scalability, security and integrity vulnerabilities, and regulatory compliance. Permissioned blockchain networks are controlled by a central authority, and only approved nodes are allowed to participate. They offer a higher level of privacy and control, making them suitable for enterprise applications, such as supply chain management, data sharing, and asset tracking. However, they face challenges such as centralization and interoperability. Hybrid blockchain networks combine the features of both public and private networks for more flexible and customizable solutions. They are suitable for apps that require openness with some level of control and privacy, such as voting systems. However, they face challenges such as complexity, interoperability, and trust.

To simplify, a permissioned blockchain network can be compared to a carefully regulated corporate intranet, whereas a permissionless network is more akin to the open and unrestricted public internet that allows anyone to join. Permissioned blockchains are typically utilized by a consortium, which is a collective of organizations and individuals working together. This is important to note as it affects other components of the blockchain [47].

### 2.3. Blockchain Data Storage, How to?

Blockchains utilize a distributed and decentralized network of computers known as nodes to store data [25,73,74,75,76,77]. The data is organized into blocks, with each block containing a collection of transactions or other pertinent information [78,79,80,81]. Blockchains employ a systematic approach to data storage, encompassing the following key elements: each block within the blockchain consists of two core components—a header and a data section. The header contains vital metadata such as a timestamp, a reference to the previous block, and a unique identifier referred to as a hash, while the data section encompasses the actual stored information or transactions. Before incorporation into the blockchain, the data section undergoes a cryptographic hashing process, yielding a distinct, fixed-size character string known as a hash. This hash, generated from the block’s data, serves the crucial role of identifying and validating the block’s integrity. Furthermore, the hashed transactions undergo a process known as Merkle tree construction. These transactions are paired and repeatedly hashed, producing a new set of hashes with each iteration. This iterative process persists until a single hash, termed the Merkle root, is attained. The Merkle tree, organized as a binary tree structure, features leaf nodes representing the hashed transactions and internal nodes representing the hashes of their child nodes. Situated in the block header, the Merkle root serves as a succinct representation and unique identifier for all transactions within the block. This allows for swift verification of the entire transaction set.

To establish a chronological order, blocks in the blockchain are linked together. Each block includes a reference (hash) to the preceding block, forming a continuous chain of interconnected blocks. This linkage mechanism ensures that any alterations to a previous block necessitate the recalculation of hashes for all subsequent blocks. In a decentralized blockchain network, multiple nodes collaborate in data validation and storage. When a new block is added, it is distributed across the network, with each participating node autonomously verifying the block’s validity through the examination of its contained hash and transactions. Various consensus algorithms are employed to reach an agreement on the block’s validity. Once a block garners validation from the network, it is appended to the individual blockchain copies maintained by each participating node. These copies undergo continuous updates and synchronization to mirror the latest blockchain state. This replication process guarantees data distribution and redundancy across numerous nodes, consequently enhancing blockchain security and resilience. Through the integration of these processes, blockchain furnishes a transparent, tamper-resistant, and verifiable approach to data storage, devoid of reliance on a central governing authority [21,47].

### 2.4. Blockchain Attacks and Mitigation

While blockchain technology offers robust security features, it is essential to acknowledge and address potential weaknesses and vulnerabilities that can emerge across various layers of the blockchain ecosystem. These include the 51% attack, primarily affecting Proof of Work (PoW) networks, where an entity controlling the majority of computational power can manipulate the blockchain. Smart contract vulnerabilities can arise from coding errors, leading to unexpected behavior or security breaches. Ensuring private key security is crucial, as compromised keys can lead to unauthorized access or tampering. Sybil attacks involve creating fake identities to disrupt the network, and network layer vulnerabilities, like DDoS attacks, can compromise connectivity. Decentralized governance can introduce governance and legal risks, impacting network security and stability. Additionally, specific attacks like the Finney attack, brute-force attack, and selfish mining attack pose distinct threats to blockchain security [57,82].

While vulnerabilities do exist in blockchain systems, many of them require substantial resources, technical expertise, or specific conditions for successful exploitation. The blockchain community actively addresses these vulnerabilities [83,84,85,86,87,88] through security research, best practices implementation, code audits, and continuous development efforts, aiming to enhance system security and robustness. Strategies to mitigate these vulnerabilities include implementing alternative consensus mechanisms like Proof of Stake (PoS) or Delegated Proof of Stake (DPoS), increasing network size and diversity, conducting smart contract audits, employing formal verification methods, ensuring robust key management through hardware wallets and multi-factor authentication, and establishing secure backup and recovery procedures. Sybil attack resistance can be achieved through reputation systems, PoS mechanisms, and identity verification protocols. Network security measures involve firewalls, traffic filtering, encryption, peer authentication, and regular security assessments. Governance and regulatory compliance are facilitated by transparent governance structures, adherence to regulations, and collaboration with regulatory bodies to align blockchain practices with legal frameworks. These proactive measures collectively contribute to bolstering blockchain security and resilience against potential threats and vulnerabilities.

## 3. Related Work

Blockchain technology has gained significant attention for its potential to revolutionize various industries, including finance, supply chain management, healthcare, and more. One area where blockchain’s impact is becoming increasingly prominent is mobile app development. Here, we explore the history and related work that highlights the integration of blockchain technology into the realm of mobile applications.

### 3.1. History

The inception of blockchain, originally conceptualized for timestamping digital documents, received considerable attention in 2008 with the introduction of Bitcoin. While the decentralized nature of Bitcoin demonstrated blockchain’s potential, by the early 2010s, visionaries recognized its broader applicability, especially in enhancing mobile app data storage security. Between 2015 and the late 2010s, as initial implementations emerged, industries with critical security requirements, like finance and healthcare, began integrating blockchain technology to protect user data. However, this surge also unveiled challenges, including scalability, energy consumption, and latency. As a response, the industry explored off-chain storage, sidechains, and sharding to surmount these hurdles. By the early 2020s, with mobile devices’ limited capabilities in focus, the emphasis shifted towards lightweight blockchain designs. Innovations, such as Zero-Knowledge Proofs, allowed for efficient data transaction validations without data exposure, further cementing blockchain’s role in fortifying mobile app data storage security. Today, as mobile apps increasingly handle sensitive information, blockchain stands as a beacon for enhanced security and user trust. Real-life examples are discussed in the coming subsection.

### 3.2. Related Work

Blockchain technology has made significant inroads into mobile payment applications, striving to establish secure, decentralized, and efficient payment solutions. Ongoing research and development endeavors aim to bolster transaction speed, trim costs, enhance security measures, and empower users with greater authority over their financial transactions. Prominent examples encompass the likes of the Samsung Blockchain Wallet [89,90]. Decentralized identity management systems for mobile apps are another critical domain where blockchain technology finds utility. These systems grant individuals greater control over their digital identities, emphasizing privacy and secure digital interactions. Notable contributions include Microsoft’s Identity Overlay Network (ION) [91], uPort, and Sovrin. The application of blockchain in supply chain management has garnered substantial attention, primarily for addressing challenges related to transparency, traceability, and efficiency. Blockchain facilitates the secure and transparent documentation of transactions along the supply chain, with each transaction meticulously time-stamped and cryptographically linked. Noteworthy initiatives include IBM’s Food Trust, Walmart’s Food Traceability Initiative [92] and Everledger [93]. Real estate also benefits from blockchain’s capacity to securely store and verify property titles and deeds in a decentralized and transparent manner, enhancing operational efficiency and transparency. An illustrative instance is the “Bitland” platform [94]. In the domain of voting systems, blockchain-based mobile apps elevate the transparency and integrity of the voting process by securely recording and verifying voting results and ballots. An example of such an application is the “Follow My Vote” platform [95].

Furthermore, blockchain technology seamlessly integrates with Internet of Things (IoT) devices to address IoT-related challenges, including data security, trust, interoperability, and data provenance [96,97,98]. Pioneering projects such as IOTA [99] utilized blockchain for IoT solutions. The secure and efficient management of digital assets, encompassing cryptocurrencies, tokens, and NFTs, represents another facet of blockchain’s impact. This includes the development of cryptocurrency wallets, NFT marketplaces, and DeFi platforms. In the healthcare sector, blockchain applications advance health data sharing and patient-centric healthcare management via mobile apps. Noteworthy instances include Medicalchain [100] and MediLedger [101], with a focus on patient control, pharmaceutical supply chain security, and healthcare data management.

These diverse applications underscore the versatility and potential of blockchain technology in catalyzing transformations across various industries, promising enhanced security, transparency, and efficiency in data management and transactions. Ongoing research and development endeavors continue to broaden the horizons of blockchain adoption within mobile applications across diverse sectors. A further examination of research gaps and unexplored areas is warranted.

### 3.3. Research Gaps and Unexplored Areas

There are several research gaps and unexplored areas that present opportunities for further investigation. Addressing the following gaps can contribute to the development of more efficient, secure, and user-friendly blockchain-based mobile applications: energy efficiency, mobile-specific consensus mechanisms, usability and user experience, scalability and performance solutions, security auditing and vulnerability assessment, cross-platform integration, integration with existing infrastructure, cost-effectiveness, data access control, offline transaction support, network connectivity and reliability, hybrid solutions, real-time data processing and data storage costs.

One specific research gap of interest is the challenge posed by data storage costs in the context of using blockchain for mobile apps [31,32,34]. While storage costs are a concern for mobile apps, research should address the overall cost-effectiveness of blockchain data storage solutions, considering transaction fees, data management expenses, and resource utilization on mobile devices, but addressing the research gap related to data storage in using blockchain for mobile app development goes beyond the monetary aspect [22,24] and focuses on optimizing the storage mechanism to accommodate the exceptional challenges posed by mobile devices, which is our focus.

The challenges related to blockchain-based data storage for mobile applications are multifaceted [102,103,104,105,106]. Firstly, mobile devices have limited storage capacity compared to traditional servers, posing difficulties for storing and accessing blockchain data, particularly when dealing with large datasets. Scalability issues within public blockchain networks become increasingly critical as mobile apps interact with and generate more data on the blockchain. Data redundancy, while essential for security and decentralization, can lead to higher storage demands, making it less practical for resource-constrained mobile devices. Ensuring efficient data access and retrieval for seamless user experiences is challenging due to blockchain’s distributed nature and potential latency. Privacy concerns arise when storing sensitive data on public blockchains, necessitating research into privacy and confidentiality solutions. Establishing data pruning mechanisms and retention policies is crucial for managing blockchain data size effectively. Additionally, energy-intensive consensus mechanisms impact the energy efficiency of mobile devices in blockchain networks, necessitating eco-friendly alternatives. Ensuring cross-platform compatibility, real-time data access, integrating off-chain storage, and addressing regulatory and compliance issues further complicate the development of blockchain-based mobile apps.

The adoption of blockchain for mobile app data storage security requires addressing these research gaps to build efficient, secure, and user-friendly solutions. By developing innovative approaches and finding solutions to these challenges, blockchain technology can offer valuable contributions to mobile app data storage and security, enhancing data integrity and privacy for a wide range of applications.

## 4. Blockchain-Based Mobile Apps Framework

### 4.1. Could Blockchain Technology Be a Solution?

Blockchain is a secure and transparent data storage technology that employs a decentralized, distributed database ledger. It works via a network of computers that validate transactions and records them using cryptographic algorithms. Because there is no central authority, the blockchain is resistant to tampering and fraud [107]. It provides a secure method of data storage and access, making it an appealing solution for a lot of applications, including supply chain management (SCM), financial transactions, and data storage. Without the need for a central authority, blockchain enables multiple parties to agree on a shared digital history [108]. The data storage mechanism provided by blockchain technology can be an innovative secure solution [109,110,111,112,113,114,115].

Implementing blockchain technology for Android data storage is not necessarily due to a problem with traditional Android data storage, but rather because blockchain technology provides certain advantages that traditional data storage systems do not. There are several reasons why the use of blockchain for data storage in Android applications should be considered [22,72,116]:

1. Decentralization and immutability: traditional Android data storage relies on centralized servers or cloud-based solutions. In contrast, blockchain offers a decentralized and distributed approach, where data is stored across multiple nodes in the network [117]. This decentralized nature ensures that data remains accessible even if some nodes fail, and it enhances data integrity and resilience against attacks. Two types of nodes are relevant here: full nodes and lightweight nodes [72,87]. When considering the integration of blockchain into mobile app development, the decision between full nodes and lightweight nodes revolves around optimizing resource efficiency and user experience. Full nodes, which form the core of blockchain networks, maintain the entire transaction history, validate transactions, and bolster security. However, they come with significant storage, computational, and bandwidth demands, rendering them impractical for mobile devices with limited resources. Conversely, lightweight nodes, engineered for efficiency and reduced resource consumption, store a minimal subset of blockchain data, synchronize swiftly, scale adeptly, and simplify user interactions. These attributes align lightweight nodes with the constraints and requisites of mobile app development. Consequently, for mobile apps seeking to harness blockchain technology, lightweight nodes stand out as the preferred choice. They ensure a smoother user experience, expedite synchronization, and enhance scalability, ultimately fostering broader blockchain adoption within the mobile app realm.
2. Tamper-resistance: data stored on a blockchain is secured using cryptographic hashing and is resistant to tampering. Once data is recorded in a block, it becomes practically immutable, ensuring that historical records cannot be altered or deleted.
3. Data trust and transparency: blockchain’s transparency and consensus mechanisms enable all participants to have visibility into the data and its history. This transparency enhances trust among users and stakeholders as they can independently verify the data.
4. Auditing and accountability: blockchain provides a permanent and auditable record of data transactions, making it valuable for auditing and maintaining a transparent record of changes [71].
5. Traceability: all transactions on a blockchain are recorded sequentially and are available for verification by any user of the network. This traceability feature is especially useful in scenarios where maintaining a verifiable record of all transactions is required.
6. Controlled access and ownership: blockchain allows for more sophisticated access control models, and users have full control and ownership of their data.
7. Interoperability and data sharing: blockchain can facilitate data sharing and interoperability between different applications or platforms, enabling seamless integration of data from multiple sources.
8. Use cases requiring consensus: certain Android applications may require consensus among multiple parties to validate and record data. Blockchain’s consensus mechanisms provide a way to achieve agreement on data validity without the need for a central authority.
9. Smart contracts for automation: blockchain technology supports smart contracts, which are self-executing contracts with predefined conditions. This automation capability can streamline processes, reduce the need for intermediaries, and improve the overall efficiency of applications.
10. Global accessibility: blockchain networks are accessible globally, making data available across borders without the need for intermediaries or regional restrictions.
  In many cases, the decision to use blockchain is driven by the requirement for decentralization, transparency, and tamper-resistant data storage, especially in scenarios where data integrity and trust are critical, such as supply chain management, medical records, financial transactions, and identity management.

Blockchain can offer several advantages over traditional encryption techniques, even with the limited memory and storage capabilities of mobile devices. In some cases, combining both technologies can be beneficial. For example, one might use encryption to secure data before it is submitted to a blockchain, ensuring its confidentiality and integrity until it becomes part of the tamper-resistant ledger. This hybrid approach leverages the strengths of both technologies to provide a more robust overall solution. The decision to use blockchain, encryption, or a combination of both depends on factors such as security requirements, decentralization needs, performance considerations, and the specific use case of a given application or system. Table 2, Table 3 and Table 4 show a comparison between blockchain solutions and traditional solutions [21,53,72,118,119,120,121], showing the new features that adopting blockchain technology can provide.

Blockchain technology and traditional encryption techniques serve distinct purposes and offer unique benefits, making each more suitable for specific scenarios. Based on the comparison table, blockchain is a preferred choice when a decentralized system for multiple parties to participate without relying on a central authority is required, making it ideal for peer-to-peer interactions, decentralized marketplaces, and multi-party collaborations. It excels in scenarios where an immutable and tamper-resistant ledger is needed to maintain transparent and auditable records of transactions or data, ensuring historical data remains unchanged. Blockchain’s secure and transparent environment is well-suited for applications that require the automated execution of agreements based on predefined rules (smart contracts). Additionally, if a mobile app involves sensitive transactions, requires an immutable and transparent record of activities, or needs to handle cryptocurrencies, digital assets, token-based systems, or decentralized digital identities, blockchain proves to be a natural fit, enhancing authentication mechanisms within mobile apps.

### 4.2. Proposed Framework Description

**Figure 2:**
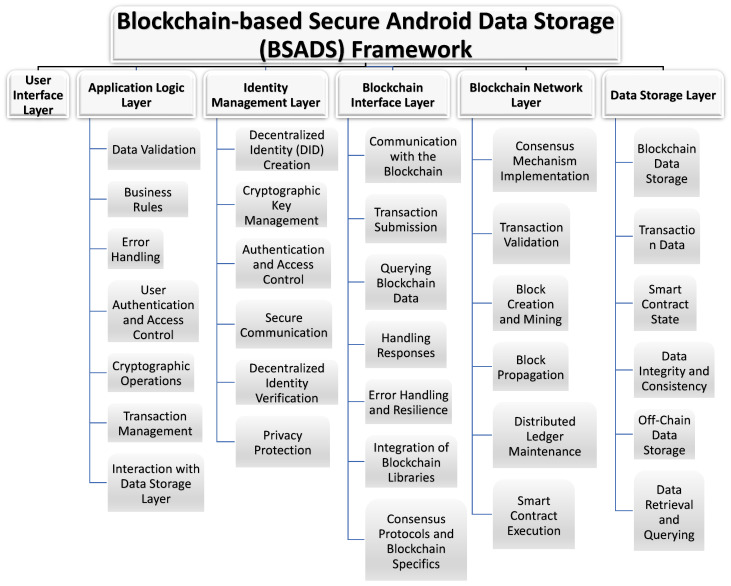
Proposed framework: Blockchain-based Secure Android Data Storage (BSADS).

A high-level model of the use of a blockchain-based system for Android data storage is suggested. We propose a six-layer framework called Blockchain-based Secure Android Data Storage (BSADS) that could help developers build a secure Android app with blockchain data storage capabilities (See Figure 2). These six layers are as follows: User Interface, Application Logic, Identity Management, Blockchain Interface, Blockchain Network and Data Storage. Each layer has a specific role and set of responsibilities within the application, and the success of each layer’s operation depends on the proper functioning of the other layers. By separating these responsibilities into different layers, the architecture becomes more modular and flexible, making it easier to manage, update, and scale the application.

The reason for dividing our proposed BSADS framework into these specific six layers is to cover the essential components and functionalities required to build a secure Android app with blockchain data storage capabilities. These six layers are considered fundamental to achieving the goals of our framework. After explaining the role of each of the six layers, and for more clarification, we chose the chatting system to be our example to show the responsibility and the flow of data through each layer.

#### 4.2.1. User Interface (UI) Layer

The User Interface (UI) Layer serves as the bridge between the application’s functionality and the end-user, providing an interface that allows users to interact with and benefit from the application’s features in a user-friendly and efficient manner.

This layer is the very visage of the application, where design and functionality come together to create a unified user experience. It includes a variety of elements, such as the layout of displays and the arrangement of buttons, as well as the selection of colors and typefaces. The UI Layer is concerned with devising interactions that are intuitive, responsive, and consistent with the app’s purpose. Whether it is a banking app, a social networking site, or an e-commerce website, the UI Layer adapts to the specific requirements of each application to ensure that users can navigate its features with ease. Developers at this layer must achieve a delicate equilibrium between aesthetics and usability as one of their primary challenges. While eye-catching visuals can attract a user’s attention, the real enchantment occurs when the interface guides them effortlessly toward their objectives. Buttons should be placed where users anticipate, menus should be straightforward, and feedback should be instantaneous. Accessibility is also of the utmost importance, ensuring that the interface is accessible to all users, including those with disabilities. Moreover, in the context of blockchain-based applications, such as those developed with the BSADS framework, the UI Layer assumes additional responsibilities. It must convey to the average user the complexities of blockchain operations, such as cryptographic key management and transaction authentication. Security prompts and sensitive data entry must be managed with the utmost care to guarantee that users can interact with the blockchain securely while enjoying a seamless user experience.

#### 4.2.2. Application Logic Layer

The Application Logic Layer is essential for maintaining the integrity and coherence of the application’s functionality and ensuring proper interactions with the underlying data storage and blockchain layers. A well-designed and efficient Application Logic Layer is important for the overall performance and user experience of the application, and it encompasses various components and responsibilities. It begins with data validation, ensuring the accuracy and consistency of user inputs and external data sources. This layer implements the core business rules and logic that govern the application’s operations, defining the necessary steps and processes aligned with use cases and requirements. Additionally, it manages error handling, ensuring user-friendly error messages for a better user experience. User authentication and access control fall within its purview, verifying user identities, granting appropriate access privileges, and enforcing access control rules, especially when dealing with sensitive data and functionalities. In blockchain-based applications, cryptographic operations, such as data signing and verification, may be integrated for data integrity and security. Furthermore, transaction management, including the creation and submission of transactions to the blockchain network, and interaction with the Data Storage Layer are handled by this layer. In some cases, it also manages application states and ensures consistency across different application components.

The Application Logic Layer powers Android apps and ensures a smooth user experience. It controls complicated business processes, tasks, and user interactions in addition to data validation. It is adaptable because it can adapt to different use cases and needs. This layer powers financial transactions, user-generated content, and e-commerce. It guarantees each process step is precise, improving application dependability and efficiency. The Application Logic Layer secures user interactions with the blockchain in blockchain-based apps. Data signing and verification are cryptographic activities to ensure data integrity and security. It manages transaction creation and submission to the blockchain network, allowing users to effortlessly use blockchain capabilities. In an age of high user expectations, the Application Logic Layer’s role in producing user-friendly error messages is crucial. It simplifies error codes into actionable information to help users fix issues. Effective error handling improves user experience and decreases irritation. This layer also authenticates and controls users to restrict access to sensitive data and actions. It protects the application from unauthorized access and data breaches, making it essential. Finally, the Application Logic Layer’s state consistency ensures a smooth user experience. This layer stabilizes data and processes regardless of application complexity.

#### 4.2.3. Identity Management Layer

The Identity Management Layer plays a pivotal role in blockchain-based applications, encompassing a range of components and responsibilities. It is responsible for managing user identities, cryptographic keys, and authentication processes within the application. This layer begins with the creation of Decentralized Identities (DIDs), offering users unique identifiers linked to the blockchain, enabling pseudonymous or anonymous interactions while maintaining identity control. This layer is also responsible for cryptographic key management, ensuring the secure generation and storage of key pairs, with public keys registered on the blockchain and private keys securely stored on users’ devices. Authentication and access control are facilitated, permitting authorized user access to specific features or data by proving ownership of a DID and cryptographic keys. Additionally, it supports secure communication through key-based message signing and verification, preserving data integrity and confidentiality. The layer can further aid in decentralized identity verification, enabling users to establish identity validity via proofs or attestations from trusted entities without divulging sensitive information. Emphasizing privacy protection, this layer allows users to control the extent of personal information shared with applications, mitigating data breach risks and unauthorized data collection, ultimately fostering a secure and privacy-respecting identity management framework.

The creation and administration of Decentralized Identities (DIDs) is one of the pillars of the Identity Management Layer. These unique identifiers, which are anchored in the blockchain, enable users to interact pseudonymously or even anonymously while retaining control over their identities. DIDs allow users to interact with applications while minimizing the disclosure of personal data, conforming to contemporary privacy expectations. In addition, this layer specializes at managing cryptographic keys. It guarantees that cryptographic key pairs are generated and stored securely, preventing unauthorized access. Public keys are recorded on the blockchain, while users’ private keys are stored on their devices. This meticulous approach to key management is essential for safeguarding user information and facilitating secure digital signatures. At the core of the Identity Management Layer are authentication and access control. It establishes the criteria for authenticating user identities and granting access privileges based on the possession of a DID and cryptographic keys. These mechanisms constitute a strong defense against unauthorized access, ensuring that only authorized users have access to particular features or data. It is essential for protecting sensitive information and sustaining the application’s integrity. This layer facilitates secure communication via key-based message signing and verification in addition to authentication. Integrity and secrecy of data are of the utmost importance, and cryptographic techniques are used to preserve these crucial aspects of secure communication. Users can have confidence that their interactions with the application are protected from inquisitive eyes and tampering attempts. In addition, the Identity Management Layer allows for decentralized identity verification. Users can establish the veracity of their identities through substantiation or attestations from trusted entities without disclosing sensitive data. This enables users to interact with blockchain ecosystems with confidence while maintaining their privacy. This layer emphasizes privacy protection and provides users with granular control over the amount of personal data shared with applications. This control reduces the likelihood of data breaches and unauthorized data collection, in accordance with contemporary privacy regulations and user expectations.

#### 4.2.4. Blockchain Interface Layer

The Blockchain Interface Layer plays a pivotal role in Android applications interacting with blockchain networks, encompassing various components and responsibilities. It is the foundation of the system that bridges the Android app with the blockchain world, facilitating secure and efficient blockchain interactions.

The layer is responsible for communicating with the blockchain network. This requires connecting blockchain nodes via protocols and APIs. The layer adjusts to the blockchain platform’s communication requirements, letting the Android app interact with the decentralized infrastructure. Additionally, the Blockchain Interface Layer excels at blockchain transaction submission. Transaction data is carefully packaged, signed using cryptographic keys, and sent to the blockchain for validation and inclusion. The integrity and validity of each transaction is crucial to blockchain-based systems. The layer lets Android apps easily query blockchain data and submit transactions. This layer effectively performs transaction status checks and blockchain data retrieval. It gives the app access to blockchain data, improving user experiences and allowing blockchain-based functionalities. However, blockchain is dynamic and difficulties can occur. By handling errors well, the layer shows its durability. It gently handles blockchain outages and transaction concerns. Despite blockchain network oscillations, its stability keeps the program running smoothly. The layer also effortlessly connects blockchain libraries or SDKs into Android apps. These libraries and SDKs facilitate blockchain development and ensure compatibility with the blockchain network’s consensus mechanisms and protocol requirements. Integration streamlines blockchain feature implementation, saving development time and resources.

#### 4.2.5. Blockchain Network Layer

The Blockchain Network Layer stands as the core element within a blockchain-based application, orchestrating the intricate dance of decentralization while safeguarding the sanctity of data. Its multifaceted role in consensus, transaction validation, and distributed ledger maintenance positions it as the linchpin of the blockchain architecture. In the quest for a robust and dependable blockchain network, seamless integration and collaboration with other layers are paramount.

Implementing the consensus method is this layer’s main task. The consensus mechanism—Proof of Work, Proof of Stake, or another—rules transaction agreement and blockchain inclusion. The whole blockchain ecosystem relies on this decision-making process for reliability and transparency. Transaction validation is crucial to the Blockchain Network Layer. It rigorously evaluates incoming transactions to verify they meet consensus processes and smart contract requirements. Blockchain integrity and security depend on this rigorous certification procedure. In Proof of Work or comparable contexts, this layer manages the complex process of mining blocks. Mining creates new blockchain blocks by solving challenging mathematical problems. The Network Layer quickly distributes mined blocks across the network, ensuring that all nodes validate and incorporate them into their blockchains. Additionally, the Blockchain Network Layer protects distributed ledger integrity. It ensures consistency across blockchain copies kept by network nodes. Consistency is essential for blockchain trust and reliability. This layer manages self-executing contracts in smart contract-enabled blockchains. It validates smart contract conditions before activation, ensuring blockchain ecosystem trust in automated contractual agreement execution.

#### 4.2.6. Data Storage Layer

The Data Storage Layer stands as the bedrock of trustworthiness and security within blockchain-based applications, underpinning the very essence of data integrity. It serves as the stalwart guardian, ensuring that information etched onto the blockchain remains impervious to tampering, creating a secure and transparent data storage solution.

Fundamentally, this layer is bestowed with the solemn responsibility of safeguarding information on the blockchain. This is achieved through the skillful arrangement of interlinked components, creating an unyielding sequence of permanence and robustness. Contained within these blocks lies a wealth of transactional data, encompassing a wide spectrum of value exchanges, as well as complex data structures that encompass a diverse range of assets and contractual arrangements. In blockchain ecosystems that adopt smart contracts, the Data Storage Layer assumes the task of preserving the up-to-date status of deployed smart contracts. The system functions as a repository for both contract-related data and the factors that dictate their functionality. The aforementioned responsibility is carried out with steadfast dedication to maintaining the integrity of the data. In order to ensure the inviolability of data, this layer utilizes the powerful capabilities of cryptographic hash functions and consensus procedures. These guardians of security remain steadfast in their efforts to prevent any retrospective endeavors to modify recorded data on the blockchain without the universal agreement of the network, hence upholding the integrity of the blockchain. In situations where the amount of data exceeds the storage capacity of the blockchain, innovative off-chain data storage methods are utilized. The proposed methods involve storing cryptographic hashes or references on the blockchain for the purpose of verification, while the primary data is stored in external databases or decentralized file systems. This prudent strategy guarantees both the ability to be easily accessed and the potential for expansion. In addition, the Data Storage Layer enhances the capability of retrieving and querying data, enabling programs to access previous data or extract specific information as needed. Aligned with the remaining five tiers of the BSADS framework, this particular tier establishes a comprehensive integration of measures pertaining to data security, user authentication, data integrity, and smooth interaction with the blockchain network. The aforementioned statement offers a solid and steadfast groundwork, poised to confront the intricacies and difficulties associated with the development of Android applications based on blockchain technology. Furthermore, it allows for flexibility and growth as circumstances demand.

These six layers collectively cover the major components and functionalities required to build a secure Android app with blockchain data storage. They establish a well-structured architecture that ensures data security, user authentication, data integrity, and seamless interaction with the underlying blockchain network. While it is possible to introduce additional layers or further divide these layers based on specific requirements [21], the provided six layers offer a strong foundation for the BSADS framework. This flexibility allows developers to adapt the framework to diverse use cases and evolving technological landscapes. As blockchain technology continues to evolve, the BSADS framework can serve as a robust starting point, offering a balance between security, performance, and scalability.

In addition, the modularity of these layers facilitates maintenance and scalability. Without rewriting the entire application, developers can enhance or extend individual layers to accommodate new features or requirements. This agility is particularly valuable in the ever-changing mobile app development landscape, where remaining ahead of the curve is crucial. The BSADS framework fosters a community of innovation and best practices by encouraging collaboration between developers and blockchain enthusiasts. By sharing insights and experiences, developers can refine and expand the framework, ensuring that it remains at the vanguard of blockchain-based Android application development. In conclusion, the BSADS framework is a landmark in the convergence of Android application development and blockchain technology. It empowers developers to create the next iteration of Android applications that leverage the transformative power of blockchain data storage by virtue of its adaptability, security, and innovation potential.

### 4.3. Flow of Data and Relationships between Layers

The user interacts with the application’s User Interface (UI), providing input or making requests (e.g., submitting a form, or initiating a transaction). Then it forwards the user’s input to the Application Logic Layer. The Application Logic Layer receives the user’s input and identifies that the user wants to perform an action that requires authentication (e.g., access a restricted feature, or sign a transaction). The layer then sends a request to the Identity Management Layer for user authentication.

The Identity Management Layer generates or retrieves the user’s decentralized identity (DID) and cryptographic key pair (public and private keys). It then uses the cryptographic keys to sign the authentication request and forwards the signed authentication request to the Blockchain Interface Layer. The Blockchain Interface Layer takes the signed authentication request and sends it to the Blockchain Network Layer to initiate the authentication process. It also verifies the authenticity of the authentication request using the user’s public key stored on the blockchain. The Blockchain Network Layer returns a response to the Identity Management Layer, confirming the successful verification of the user’s authentication request.

The Identity Management Layer informs the Application Logic Layer that the user’s authentication is successful, and the user is authorized to perform the requested action. Based on the user’s input and authentication status, the Application Logic Layer decides whether to read or write data to the Data Storage Layer. If the user requests to read data, the Application Logic Layer sends a query to the Data Storage Layer. For on-chain data, the Data Storage Layer interacts with the Blockchain Interface Layer to fetch the requested data from the blockchain (e.g., reading user account balance from a smart contract). For off-chain data, the Data Storage Layer accesses external databases or decentralized file systems using appropriate APIs or protocols (e.g., IPFS). If the requested data is on-chain, the Blockchain Interface Layer sends a query to the Blockchain Network Layer to retrieve the relevant information.

The Blockchain Network Layer returns the requested data to the Data Storage Layer and the Data Storage Layer forwards the retrieved data to the Application Logic Layer. The Application Logic Layer then processes the retrieved data, performs any necessary calculations or manipulations, and prepares the data to be displayed. It sends the processed data back to the UI Layer. Finally, the UI Layer displays the processed data to the user or provides appropriate feedback based on the user’s actions and the results of the requested operation. The relationship between the different layers of the proposed framework is best described as sequential and dependent. This means that one layer’s output is often the input for the next layer, and each layer relies on the preceding layers to perform its functions properly. However, the actual flow of data and interactions between the layers is bidirectional, allowing for a dynamic exchange of information to create a fully functional and secure app using blockchain technology. The bidirectional relationships ensure that each layer can communicate with others as needed, leading to a cohesive and well-integrated application architecture.

The complex interplay of data within the BSADS framework serves as a prime illustration of the seamless collaboration among its various layers. Although the progression of data may seem to follow a linear pattern, it also demonstrates an intrinsic capacity for flexibility, enabling it to accommodate differences and adapt to particular applications. The aforementioned dynamic interaction not only serves to guarantee the security and integrity of data, but also facilitates a smooth and uninterrupted user experience. Fundamentally, the interconnections among the various levels can be metaphorically compared to a meticulously coordinated symphony, wherein each musical instrument assumes a crucial function in the composition of a harmonious entirety. Similar to the role of a conductor in guiding musicians to create harmonious music, the BSADS framework serves as a guiding framework for managing the flow of data in order to develop secure and efficient Android applications that incorporate blockchain functionalities. The success of the framework is rooted in the fundamental synergy that exists between its levels, which empowers developers to create cutting-edge and dependable applications tailored for the contemporary environment.

### 4.4. Illustration of the Data Flow in the Context of Chatting Application

#### 4.4.1. User Interface (UI) Layer: Displaying Chat Messages

The UI Layer of the chatting application captures user input, displays chat messages, and facilitates the real-time display of messages in the chatroom.The UI Layer receives the updated chat history from the Application Logic Layer. It displays the chat messages, including both incoming and outgoing messages, to the user in a readable format. The user can view the chat history and continue interacting with the chatting application. Moreover, within the ever-changing realm of chat applications, it is imperative for the UI Layer to adjust in accordance with user preferences and accessibility needs. It is imperative to give precedence to designs that are both clean and user-friendly, while also ensuring support for multimedia messaging and incorporating accessibility elements. The use of a user-centric approach is essential for maintaining the competitiveness and inclusivity of the chat application, hence expanding its reach to a wider user base.

#### 4.4.2. Application Logic Layer: Data Retrieval and Display

The Application Logic Layer validates user inputs, processes chat messages, and handles user authentication requests. If the user is not authenticated, it triggers the Identity Management Layer for verification. It validates the message to ensure it meets the required criteria (e.g., not empty, within character limits). The Application Logic Layer prepares the chat message for submission to the blockchain, which may include adding metadata such as timestamps or user identifiers. It retrieves the updated chat history from the Data Storage Layer in response to real-time updates or user queries. It processes the data and prepares it for display in the UI Layer.

#### 4.4.3. Identity Management Layer: Signing and Verification

The Identity Management Layer verifies user identities using decentralized identity solutions, such as DIDs and cryptographic keys. The Identity Management Layer signs chat messages with the user’s private key to ensure message integrity. It may also verify the authenticity of received messages from other users using their public keys. Moreover, within the domain of identity management, the crucial factor lies in the ongoing improvement. The incorporation of biometric and multi-factor authentication methods, along with the adoption of evolving authentication standards, has the potential to enhance the robustness of user identity verification within the chat application, hence bolstering security measures.

#### 4.4.4. Blockchain Interface Layer: Transaction Submission

The Blockchain Interface Layer receives the signed chat message from the Identity Management Layer. It communicates with the blockchain network using appropriate protocols (e.g., HTTP, WebSocket) and APIs. The Blockchain Interface Layer packages the chat message as a transaction and submits it to the blockchain for validation and inclusion in the blockchain. In the Blockchain Interface Layer, efficiency and compatibility are key. Using appropriate protocols and APIs, this layer should communicate with the blockchain network seamlessly. Developers should optimize transaction packaging and submission to reduce latency and resource usage to improve functionality. For effective transaction validation and inclusion, blockchain technologies and protocols must be updated. The Blockchain Interface Layer can speed up chat message submission to the blockchain network and ensure its timely validation and inclusion by focusing on these enhancements.

#### 4.4.5. Blockchain Network Layer: Transaction Validation and Recording

The Blockchain Network Layer receives the submitted chat message transaction from the Blockchain Interface Layer, including user identities and signed chat messages. It validates the transaction to ensure that the digital signature is valid and that the user has the authority to submit the message. If the transaction is valid, the Blockchain Network Layer records the chat message in a new block in the blockchain. The chat message becomes a permanent and immutable part of the distributed ledger. The Blockchain Network Layer sends real-time updates to the Blockchain Interface Layer after the chat message is successfully added to the blockchain. The Blockchain Interface Layer processes these updates and informs the Application Logic Layer.

#### 4.4.6. Data Storage Layer: Chat Message Storage

The Data Storage Layer is responsible for securely storing chat messages and user identity information on the blockchain. Chat messages are stored in blocks linked together to form an immutable and tamper-resistant chain. User identity information, such as cryptographic keys, is also securely recorded on the blockchain. This data flow demonstrates how chat messages are securely transmitted from the User Interface Layer to the Blockchain Network Layer for validation and storage. The use of blockchain technology ensures that chat messages are tamper-resistant, immutable, and transparent, enhancing the security and trustworthiness of the chatting application.

We are sure that the actual implementation may involve more complexity, depending on the chosen blockchain platform and specific requirements and limitations. This framework serves as a conceptual guide, designing the "best" model for the (BSADS) framework would depend on various factors, including the specific requirements, use cases, and constraints of the application. We suggested a high-level (generalized) architectural model that incorporates the main components of the BSADS framework and provides a robust and scalable solution.

### 4.5. Challenges and Optimization Techniques

While blockchain technology holds promise for various applications, there have been instances of unsuccessful implementations and notable challenges [122]. Some unsuccessful examples include mobile voting projects that faced issues like low voter turnout, technical glitches, and security vulnerabilities, exemplified by West Virginia’s 2018 pilot criticized for lacking security and transparency due to reliance on a centralized server without end-to-end encryption [123]. Ethereum Name Service (ENS) encountered scalability and performance challenges as the demand for domain names exceeded its capacity, causing delays and user difficulties [124]. The Parity Wallet, a popular Ethereum wallet, suffered a critical vulnerability that allowed an attacker to freeze and lock users’ funds, eroding trust [125]. Additionally, the DAO (Decentralized Autonomous Organization) hack in 2016 resulted in the theft of funds, leading to a hard fork of the Ethereum blockchain, highlighting vulnerabilities that can impact blockchain’s security and data integrity [126]. These examples show that while blockchain can offer significant benefits for data storage, it is not a magic solution, and it is important to carefully consider the specific requirements and constraints of each app before deciding to use it. It is also important to note that there may be one successful implementation of blockchain in, e.g., voting systems, and at the same time there may be an unsuccessful one elsewhere; this highlights that programmers still lack the standardization for deploying the technology, and this has led us to consider how to suggest a solution to that issue.

The performance of blockchain-based data storage systems is limited by a number of factors, including the number of nodes in the network, the block size and the network bandwidth. These factors can lead to bottlenecks and delays in the processing of blockchain transactions [122]. On the other hand, the scalability is also limited by a number of factors, including: the throughput of the underlying blockchain network, the size of the blockchain ledger and the consensus mechanism. These factors can limit the number of transactions that can be processed per unit of time. Performance and scalability are closely related because a system with good performance is more likely to be scalable since it can efficiently handle a higher volume of tasks or data without encountering bottlenecks or excessive resource consumption. The relationship between performance and scalability in blockchain-based mobile apps is interconnected, but achieving both simultaneously can be challenging. However, these limitations are not insurmountable, and there are a number of techniques that can be used to improve them. As the technology matures, it is likely that these limitations will be addressed by researchers in the future; this study is one that can help solve the problem [127,128,129,130,131].

Several factors contribute to the limitations faced by blockchain-based mobile applications [22,132]. First, data storage and retrieval present significant challenges. The ever-expanding size of the blockchain, referred to as blockchain bloat, can overwhelm mobile devices with limited computing capabilities. Additionally, blockchain’s restricted throughput can result in slower transaction times for extensive data, and latency in data retrieval may not meet mobile app speed requirements. The associated costs of storing data on the blockchain, including transaction fees and storage expenses, can strain budgets. Privacy and security concerns stem from blockchain transparency, potentially granting unauthorized access to sensitive data. Complex smart contracts with large datasets can impose resource-intensive computational and storage costs on mobile apps, affecting cost-effectiveness. Second, insufficient consideration of mobile-specific constraints, such as limited computational power, memory, storage, and network bandwidth, can lead to suboptimal user experiences, including battery drain and slow performance. Finally, different blockchain consensus algorithms, like Proof of Work (PoW) and Proof of Stake (PoS), have unique scalability and performance implications. PoW’s computational intensity strains mobile devices, causing slower processing and higher energy consumption, while PoS introduces centralization risks and economic barriers to participation in mobile apps.

A thorough understanding of these limitations can assist developers in selecting the most appropriate choice to meet the specific needs of mobile applications, ultimately achieving the desired scalability and performance. In what follow, we present some blockchain-focused technical optimization strategies to tackle these challenges so that the stakeholders should not be dissuaded from using the technology [133,134,135,136,137].

First, blockchain pruning [138,139,140] selectively discards older blocks, significantly reducing storage requirements, enhancing scalability, and enabling lightweight nodes [141,142,143,144,145,146] to join the network. Additionally, lightweight nodes prove useful for mobile apps by conserving device resources. Exploring off-chain solutions [147,148,149,150] for non-critical or large-volume data transactions can improve performance and scalability without overburdening the main blockchain. Low-energy consensus algorithms [151,152,153,154], such as Proof of Authority and Delegated Proof of Stake, are vital for resource-efficient mobile app development. Smart transaction prioritization mechanisms [155] optimize transactions based on various criteria, improving the user experience. User-friendly interfaces, batch processing, real-time data updates, and optimized smart contracts streamline blockchain interactions, minimize latency, and enhance security. Load balancing [156,157,158], device compatibility testing and hybrid approaches combining on-chain and off-chain operations provide balanced advantages for blockchain-enabled mobile apps. Lazy loading [159] conserves resources, and effective local storage management ensures efficient data retrieval. Employing cryptographic libraries and hardware acceleration can improve cryptography performance. Sharding [136,160], a scalability technique, divides the blockchain network into smaller shards, offering concurrent transaction processing and enhanced scalability. Finally, proactive error handling provides users with informative feedback and troubleshooting tips for a seamless experience.

By adopting these blockchain-specific optimization strategies, developers can enhance the performance and scalability of Android mobile applications using blockchain technology. But something to be kept in mind is that the implementation of these strategies will vary depending on the specific blockchain network, the application’s requirements, and the programming languages and frameworks used.

## 5. Future Directions and Recommendations

In the world of blockchain and mobile app data storage security, several recommendations and future directions emerge. Firstly, leveraging blockchain’s distributed storage mechanisms to offload data storage burdens from mobile devices by employing lightweight nodes, such as SPV wallets, can enhance resource efficiency while conserving device resources. However, developers must weigh the trade-offs between security, decentralization, and resource efficiency based on specific use cases and requirements. Secondly, addressing scalability concerns through ongoing research and development efforts remains essential. Future endeavors should concentrate on devising novel solutions to enhance the scalability of blockchain-based data storage in mobile apps. Thirdly, exploring the integration of blockchain with emerging technologies like AI and IoT offers opportunities for creating advanced mobile apps that harness the strengths of both blockchain and these technologies. Fourthly, improving the user experience and adoption of blockchain-based data storage requires user-friendly interfaces and seamless integration with emerging technologies. Staying informed about regulatory developments and ensuring compliance is paramount. Furthermore, examining blockchain’s applicability across various sectors and contexts and customizing solutions to meet specific industry needs is a promising avenue. Enhanced security measures, decentralization, and increased adoption among developers and users are also crucial. Formal methods, artificial intelligence, and robust testing and verification techniques can fortify the reliability and security of blockchain systems and data storage in mobile apps.

Furthermore, it is crucial to investigate the possible synergistic effects that may arise from the integration of blockchain technology and quantum computing. The advancement of quantum computing presents both obstacles and opportunities in the realm of blockchain security. The primary area of investigation should prioritize the advancement of cryptographic algorithms that are resistant to quantum computing, as well as the examination of strategies for effectively incorporating these algorithms into blockchain networks. The use of this proactive strategy can effectively safeguard the durability and sustainability of blockchain-powered data storage in light of potential quantum-related risks. In addition, it is imperative to emphasize the significance of fostering collaboration among academics, developers, and policymakers in order to effectively construct universally accepted security procedures and regulations for the implementation of blockchain technology in the realm of quantum computing. By effectively tackling these growing difficulties, the blockchain community may persistently develop and adjust to the dynamic environment of data storage security in mobile applications.

## 6. Conclusions

In conclusion, blockchain-based data storage holds significant potential for enhancing the security and reliability of mobile apps. Its decentralized structure, distributed ledger, consensus, security and immutability, durability and availability, transparency, scalability, efficiency, smart contracts, encryption, access control mechanisms, decentralized storage systems, and reputation system offer several benefits over traditional centralized data storage solutions. However, there are also several challenges associated with the adoption and integration of blockchain-based data storage in mobile apps, such as scalability, performance and cost, complexity and management, compatibility and integration, limited use cases, and limited adoption.

In the context of mobile app development, lightweight nodes are generally more suitable due to their lower resource requirements and faster synchronization. Full nodes might be too resource-intensive for most mobile apps, potentially leading to a poor user experience and high data consumption. In terms of data storage security, full nodes offer a higher level of security due to their direct verification of all transactions and blocks. They ensure the authenticity of the entire blockchain history, making them the most reliable source for accurate data. In contrast, lightweight nodes compromise a degree of security to achieve better efficiency by depending on external validation sources. This strategy is appropriate in situations where limitations in resources are a consideration, although it introduces a need for reliance on the reliability of the data source.

Overall, blockchain-based data storage in mobile apps is still an emerging technology, and its full potential is yet to be realized. However, with ongoing research and development, and increasing adoption, it has the potential to revolutionize the way data is stored, managed, and shared in mobile apps, and provide a more secure, transparent, and reliable solution for users and developers alike.

## Figures and Tables

**Table 1:** Types of blockchain.

BC Net Type	Details	Benefits	Usage	Main Challenges
Permissionless OR Public OR Open	• They are decentralized (not controlled by others). • Transactions are validated by a network of nodes through a consensus mechanism. • They are transparent (not anyone can view and verify the transactions that occur on the network).	• Offer transparency and immutability (once a transaction is recorded, it cannot be altered or deleted). • Offer a high level of security, as it is protected by cryptography and a network of nodes. • Accessible to anyone without the need for a third party.	• Used for decentralized apps, such as Bitcoin. • Used for apps that require transparency, security, and decentralization, such as smart contracts and supply chain management. Ethereum is an example that allows developers to build such apps.	• Scalability: as the number of transactions on the network increases, the time and cost of processing those transactions can also increase, leading to longer wait times and higher fees. • Security and Integrity: they are vulnerable to 51% attacks or DoS attacks. • Regulatory: it is difficult to comply with regulatory requirements, such as know-your-customer.
Permissioned OR Private OR Closed	• Controlled by a central authority, which determines who can access the network. • Transactions are validated by a network of nodes, but only approved nodes are allowed to participate. • Not transparent, only approved participants can verify the transactions.	• Offer a higher level of privacy and confidentiality, as only approved participants can access the network. • Offer a higher level of control, as the central authority can set the rules. • Can be customized to suit the needs of specific use cases, such as SCM.	• Commonly used for enterprise applications, such as SCM, data sharing, and asset tracking. • They can also be used for secure communication and collaboration between organizations and parties. • Examples: Hyperledger Fabric and Quorum.	• Centralization: this can weaken trust and transparency and limit the participation of other parties. • Interoperability: as they may be operating on different platforms or standards for data and transactions, it can be difficult to integrate and share data between different private blockchain networks.
Hybrid	• Combine the features of public and private networks for a more flexible and customizable solution. • They can be open or closed to anyone, depending on the needs of the app. • Transactions are validated by a network of nodes through a consensus.	• Offer the benefits of both networks, such as transparency and security, as well as privacy and control. They are more flexible and customizable than either public or private networks alone, allowing for a wider range of apps and use cases.	• Used for apps that require openness with some level of control and privacy. • Used for voting systems, where transparency and security are important, but where privacy and control are also necessary to prevent fraud and manipulation. • For example: Dragonchain.	• Complexity: it combines the features of both. This can make it more difficult to develop, maintain, and secure apps on the network. • Interoperability: it is difficult to integrate and share data between different networks. • Trust: trust is required in components of both networks. The public may be subject to attacks, while the private may be subject to centralization and control.

**Table 2:** Blockchain vs. traditional encryption techniques.

Aspect	Traditional Techniques	Blockchain
Scalability and Performance	• Does not involve a consensus process, so there is no need for resource-intensive consensus mechanisms. • Does not involve smart contracts and does not incur execution costs associated with blockchain-based smart contract platforms.	• Consensus mechanisms require energy consumption and computational resources, impacting the overall overhead of the system. • Executing smart contracts can require payment of transaction fees, particularly for complex or resource-intensive operations.
Decentralization	• The data and encryption keys are typically managed by a centralized entity or stored in a centralized server. • Data management and access control are under the control of a central authority.	• Inherently provides decentralization. This decentralized nature can improve resilience and reduce single points of failure. • Participants collectively manage data and reach consensus. • By utilizing blockchain’s distributed storage mechanism, mobile apps can store data on the blockchain network, offloading the storage burden from the device itself. • Blockchain’s use of cryptographic techniques, such as hashing and digital signatures, enhances the security of data stored on the blockchain. Even if an attacker gains access to a single node or compromises data on one node, the distributed nature of the blockchain makes it difficult for them to alter the data across the entire network. • In a blockchain network, data is replicated and distributed across multiple nodes. Each node maintains a copy of the entire blockchain, and consensus mechanisms ensure that changes to the data are validated by the network. This decentralized approach reduces the risk of insider threats as no single entity has complete control over the data.
Consensus	• Does not involve selecting a consensus mechanism as it is a process applied within the app or on the server side. • Does not inherently address fault tolerance mechanisms for ensuring system resilience in the event of failures or attacks. • Does not involve community-based consensus mechanisms for governance decisions. • Does not involve distributed consensus mechanisms since it focuses on securing data.	• Developers need to choose an appropriate consensus mechanism based on the specific use case and desired level of decentralization. • Often incorporate fault tolerance measures, making the network more resistant to adversarial attacks and system failures. • Rely on community consensus for protocol upgrades and governance decisions, making it a collaborative and decentralized process. • Distributed consensus mechanisms are crucial for reaching agreement among network participants, ensuring consistency and trust. • Since blockchain operates on a consensus mechanism, any proposed changes to the blockchain must be agreed upon by the network. This consensus process helps in detecting and preventing unauthorized changes, including those resulting from zero-day exploits. It provides an additional layer of protection against unknown vulnerabilities.
Transparency and Auditing	• Does not offer the same level of auditability and transparency as blockchain. Access to encrypted data can be challenging to monitor without additional logging mechanisms. • Does not inherently address the governance transparency and decision-making processes that are essential in decentralized systems. • Emphasizes data confidentiality, and data remains opaque to unauthorized users.	• Blockchain’s transparent ledger allows for easy verification of data integrity and a comprehensive audit trail of all transactions. • Blockchain’s decentralized governance allows for transparency in decision making and community-driven updates to the system. • Blockchain balances data transparency and confidentiality, as public blockchains are transparent to all participants, while private blockchains allow for controlled data access. • Can be applied to various use cases, such as supply chain management, identity verification, and financial transactions, providing an audit trail for data operations and ensuring data integrity in a transparent and trustless manner.

**Table 3:** Blockchain vs. traditional encryption techniques.

Aspect	Traditional Techniques	Blockchain
Data Immutability	• Encryption allows for data modification and updates when authorized users possess the decryption keys. Data can be changed or deleted as needed.	• Data once recorded on the blockchain cannot be easily modified, which can be both an advantage and a limitation depending on the use case.
Smart Contract and Automation	• Does not inherently support smart contract functionality. Smart contracts are not directly relevant to encryption techniques. • Does not facilitate dispute resolution or automated execution of contractual terms as smart contracts do.	• Smart contracts enable developers to build complex decentralized applications that can automate actions. • Can automatically execute predefined conditions, streamlining dispute resolution and contractual agreements.
Data Recovery and Backup	• Can offer straightforward data recovery and backup processes. Backing up encrypted data securely allows for easy restoration in case of data loss.	• It provides data integrity through immutability, but recovery and backup of data might be more complex, especially in public blockchains where data cannot be deleted.
Cross-Platform Compatibility	• Traditional encryption techniques are generally cross-platform compatible and can be implemented on various operating systems and devices.	• Integrating blockchain into mobile apps might require additional considerations for cross-platform compatibility, especially when using different blockchain platforms or protocols.
Data Sharing Control	• May not inherently support granular data permissions and fine-grained sharing options. Data access controls often need to be managed separately. • May require additional mechanisms for secure data sharing and collaboration among multiple users.	• Blockchain platforms can implement smart contracts and access control mechanisms, enabling fine-grained data permissions and sharing based on predefined rules. • Blockchain’s decentralized nature can facilitate secure data sharing and collaboration among network participants, particularly in permissioned blockchains.
Real-Time Data Processing	• Does not inherently provide data monotonicity or cryptographic integrity proofs, which can be essential in certain use cases.	• Blockchain’s immutability ensures data monotonicity, and cryptographic proofs can be used to verify data integrity.
Monetization of Data	• Does not inherently support data monetization models or mechanisms for value exchange within the application.	• Blockchain enables the creation of token economies and smart contracts, providing opportunities for data monetization and value exchange.
Data Anonymization and Pseudonymization	• Traditional encryption may not inherently provide data anonymization or pseudonymization capabilities, which can be important for privacy-sensitive applications.	• Blockchain platforms can enable data anonymization or pseudonymization through techniques like smart contracts or cryptographic hashing.
Data Governance Models	• Does not directly enforce data governance models beyond basic access controls.	• Can enforce predefined data governance models through smart contracts and community consensus.
Cross-Chain Interoperability	• Encryption does not inherently address cross-chain interoperability, as it is primarily focused on securing data within a specific system.	• Achieving cross-chain interoperability between different blockchain networks presents technical challenges but is being actively explored in the blockchain space.
Integration with External Oracles and Real-World Data Sources	• May not inherently involve integration with external oracles or real-world data sources for data verification.	• Blockchain-based applications can use external oracles to verify real-world data and trigger smart contract execution based on off-chain events.
Regulatory Requirements for Data Breach Notifications	• Can help meet certain regulatory requirements for data breach notifications by safeguarding sensitive information. • Traditional encryption techniques are well-established and often comply with various data protection regulations and industry standards.	• In the case of a data breach on a blockchain network, the immutable nature of the data may create challenges in complying with data breach notification regulations. • Blockchain’s decentralized and immutable nature might pose challenges in meeting certain regulatory requirements, especially regarding the "right to be forgotten" and data deletion.

**Table 4:** Blockchain vs. traditional encryption techniques.

Aspect	Traditional Techniques	Blockchain
Smart Contracts for Access Control	• No smart contracts: to control access to encrypted data, a symmetric or asymmetric key-based encryption scheme is typically used. • In symmetric encryption, to grant access to specific data, the user would need to securely share the encryption key with other authorized users. This approach is not granular, as all users with the encryption key will have access to all encrypted data. • In asymmetric encryption, data encrypted with a recipient’s public key can only be decrypted using their corresponding private key. This allows for finer-grained access control, as one can encrypt data with different users’ public keys, limiting access to only those with the corresponding private keys.	• While known encryption techniques can provide access control and data protection in mobile apps, smart contracts on a blockchain offer additional benefits, making them a powerful tool for managing fine-grained access control policies for sensitive data in decentralized and transparent applications. • Smart contracts operate on a transparent and publicly auditable blockchain, ensuring that access control rules are visible to all stakeholders. • Once deployed on the blockchain, its code and rules cannot be altered, ensuring the consistency, immutability and integrity of access control policies. • They are executed on a decentralized network of nodes, removing the need for a centralized authority to manage access control. • They are automatically enforcing access control rules based on predefined conditions, reducing the reliance on manual configurations. • While known encryption techniques can provide access control and data protection in mobile apps, smart contracts on a blockchain offer additional benefits of transparency, immutability, decentralization, and automation, making them a powerful tool for managing fine-grained access control policies for sensitive data in decentralized and transparent applications.
Data Integrity	• Encryption does not alone address the trustworthiness and integrity of the data itself. To address data integrity through encryption, additional measures like data authentication, checksums, hashing, access controls, and auditing need to be implemented alongside.	• Blockchain, with its decentralized and tamper-evident nature, provides a means to establish trust and ensure data integrity. By storing data on the blockchain, mobile apps can benefit from the inherent security properties of blockchain technology.
Key Management	• Storing and protecting encryption keys on mobile devices can be vulnerable to attacks such as key extraction or unauthorized access. • It also offers robust key management practices through symmetric key management, asymmetric key pair management, secure key exchange, and key expiry and revocation.	• Blockchain can address this challenge by utilizing decentralized key management mechanisms. Keys can be securely stored on the blockchain, reducing the risk of key compromise and enhancing overall key management security. • Smart contracts can enforce access control policies for encryption keys to ensure that only authorized entities can use the keys. • It can facilitate key recovery mechanisms through smart contracts or multi-signature setups. In case of key loss, users can use predefined procedures to regain access to their encrypted data. • It eliminates the need for users to trust a central authority with their keys by relying on consensus mechanisms. • It can be used for secure and transparent key distribution between parties, ensuring that the right keys are provided to the right individuals or devices. • While blockchain can enhance key management by offering decentralized key storage, trustless access control, and key recovery mechanisms, it is not a replacement for encryption. Blockchain’s primary role is not data encryption but rather decentralized and secure record-keeping, while encryption focuses on data confidentiality and integrity.

## References

[1] de Oliveira G.A., Oliveira O.d.F., de Abreu S., de Bettio R.W., Freire A.P. (2022). Opportunities and accessibility challenges for open-source general-purpose home automation mobile applications for visually disabled users. Multimed. Tools Appl..

[2] Soodan V., Jamwal M., Rana N.P., Sharma D., Chakraborty S. (2023). Modelling the adoption of agro-advisory mobile applications: A theoretical extension and analysis using result demonstrability, trust, self-efficacy and mobile usage proficiency. J. Agribus. Dev. Emerg. Econ..

[3] Krichen M. (2021). Anomalies detection through smartphone sensors: A review. IEEE Sens. J..

[4] Zaina L.A., Fortes R.P., Casadei V., Nozaki L.S., Paiva D.M.B. (2022). Preventing accessibility barriers: Guidelines for using user interface design patterns in mobile applications. J. Syst. Softw..

[5] Galetsi P., Katsaliaki K., Kumar S. (2023). Exploring benefits and ethical challenges in the rise of mHealth (mobile healthcare) technology for the common good: An analysis of mobile applications for health specialists. Technovation.

[6] Krichen M. (2023). Convolutional neural networks: A survey. Computers.

[7] Alkhudaydi O.A., Krichen M., Alghamdi A.D. (2023). A Deep Learning Methodology for Predicting Cybersecurity Attacks on the Internet of Things. Information.

[8] Chandran V.P., Balakrishnan A., Rashid M., Pai Kulyadi G., Khan S., Devi E.S., Nair S., Thunga G. (2022). Mobile applications in medical education: A systematic review and meta-analysis. PLoS ONE.

[9] Suchodolska G., Senkus E. (2022). Mobile applications for early breast cancer chemotherapy-related symptoms reporting and management: A scoping review. Cancer Treat. Rev..

[10] Garg S., Baliyan N. (2021). Comparative analysis of Android and iOS from security viewpoint. Comput. Sci. Rev..

[11] Muhammad Z., Anwar Z., Javed A.R., Saleem B., Abbas S., Gadekallu T.R. (2023). Smartphone Security and Privacy: A Survey on APTs, Sensor-Based Attacks, Side-Channel Attacks, Google Play Attacks, and Defenses. Technologies.

[12] Balapour A., Nikkhah H.R., Sabherwal R. (2020). Mobile application security: Role of perceived privacy as the predictor of security perceptions. Int. J. Inf. Manag..

[13] Abdullah H., Zeebaree S.R. Android Mobile Applications Vulnerabilities and Prevention Methods: A Review. Proceedings of the 2021 2nd Information Technology to Enhance e-Learning and Other Application (IT-ELA).

[14] Tovino S.A. (2020). Privacy and security issues with mobile health research applications. J. Law Med. Ethics.

[15] Lin W., Xu M., He J., Zhang W. (2021). Privacy, security and resilience in mobile healthcare applications. Enterp. Inf. Syst..

[16] Yanholenko O., Cherednichenko O., Yakovleva O., Arkatov D. A Model for Estimating the Security Level of Mobile Applications: A Fuzzy Logic Approach. Proceedings of the Intel ITSIS.

[17] Antonishyn M., Misnik O. Analysis of testing approaches to Android mobile application vulnerabilities. Proceedings of the ITS.

[18] Weichbroth P., Łysik Ł. (2020). Mobile security: Threats and best practices. Mob. Inf. Syst..

[19] Patel V., Khatiwala F., Shah K., Choksi Y. (2020). A review on blockchain technology: Components, issues and challenges. Proceedings of the ICDSMLA 2019: Proceedings of the 1st International Conference on Data Science, Machine Learning and Applications.

[20] Michael J., Cohn A., Butcher J.R. (2018). Blockchain Technology.

[21] Zeng S.Q., Huo R., Huang T., Liu J., Wang S., Feng W. (2020). Survey of blockchain: Principle, progress and application. J. Commun..

[22] Viriyasitavat W., Hoonsopon D. (2019). Blockchain characteristics and consensus in modern business processes. J. Ind. Inf. Integr..

[23] Lahami M., Maâlej A.J., Krichen M., Hammami M.A. (2022). A Comprehensive Review of Testing Blockchain Oriented Software. ENASE.

[24] Morkunas V.J., Paschen J., Boon E. (2019). How blockchain technologies impact your business model. Bus. Horizons.

[25] Guo H., Yu X. (2022). A Survey on Blockchain Technology and its security. Blockchain Res. Appl..

[26] Lopes E.J., Kataria S., Keshav S., Ikram S.T., Ghalib M.R., Shankar A., Krichen M. (2022). Live video streaming service with pay-as-you-use model on Ethereum Blockchain and InterPlanetary file system. Wirel. Netw..

[27] Tarwireyi P., Terzoli A., Adigun M.O. (2022). BarkDroid: Android malware detection using bark frequency Cepstral coefficients. Indones. J. Inf. Syst..

[28] Chart: Android Is the Most Vulnerable Operating System|Statista. https://www.statista.com/chart/7478/android-is-the-most-vulnerable-operating-system/.

[29] Poonguzhali P., Dhanokar P., Chaithanya M., Patil M.U. (2016). Secure storage of data on android based devices. Int. J. Eng. Technol..

[30] Top 7 Mobile Security Threats. https://www.kaspersky.com/resource-center/threats/top-seven-mobile-security-threats-smart-phones-tablets-and-mobile-internet-devices-what-the-future-has-in-store.

[31] Fredj O.B., Cheikhrouhou O., Krichen M., Hamam H., Derhab A. (2021). An OWASP top ten driven survey on web application protection methods. Proceedings of the Risks and Security of Internet and Systems: 15th International Conference, CRiSIS 2020, Paris, France, 4–6 November 2020.

[32] Acharya S., Ehrenreich B., Marciniak J. OWASP inspired mobile security. Proceedings of the 2015 IEEE International Conference on Bioinformatics and Biomedicine (BIBM).

[33] Nagarjun P., Ahamad S.S. (2018). Review of Mobile Security Problems and Defensive Methods. Int. J. Appl. Eng. Res..

[34] OWASP Mobile Top 10|OWASP Foundation. https://owasp.org/www-project-mobile-top-10/.

[35] Mobile Application Security: 2021’s Breaches. https://www.darkreading.com/application-security/mobile-application-security-2021-s-breaches.

[36] Razgallah A., Khoury R., Hallé S., Khanmohammadi K. (2021). A survey of malware detection in Android apps: Recommendations and perspectives for future research. Comput. Sci. Rev..

[37] Altuwaijri H., Ghouzali S. (2020). Android data storage security: A review. J. King Saud-Univ.-Comput. Inf. Sci..

[38] Nakamoto S.B. (2018). A Peer-to-Peer Electronic Cash System. https://bitcoin.org/bitcoin.pdf.

[39] Jabbar R., Dhib E., Said A.B., Krichen M., Fetais N., Zaidan E., Barkaoui K. (2022). Blockchain technology for intelligent transportation systems: A systematic literature review. IEEE Access.

[40] Namane S., Ben Dhaou I. (2022). Blockchain-Based Access Control Techniques for IoT Applications. Electronics.

[41] Krichen M., Ammi M., Mihoub A., Almutiq M. (2022). Blockchain for modern applications: A survey. Sensors.

[42] Jabbar R., Krichen M., Kharbeche M., Fetais N., Barkaoui K. (2020). A formal model-based testing framework for validating an IoT solution for blockchain-based vehicles communication. ENASE.

[43] Gupta M., Kumar R., Shekhar S., Sharma B., Patel R.B., Jain S., Dhaou I.B., Iwendi C. (2022). Game theory-based authentication framework to secure internet of vehicles with blockchain. Sensors.

[44] Abbas A., Alroobaea R., Krichen M., Rubaiee S., Vimal S., Almansour F.M. (2021). Blockchain-assisted secured data management framework for health information analysis based on Internet of Medical Things. Pers. Ubiquitous Comput..

[45] Jabbar R., Fetais N., Kharbeche M., Krichen M., Barkaoui K., Shinoy M. (2021). Blockchain for the internet of vehicles: How to use blockchain to secure vehicle-to-everything (v2x) communication and payment. IEEE Sens. J..

[46] Moulahi T., Jabbar R., Alabdulatif A., Abbas S., El Khediri S., Zidi S., Rizwan M. (2023). Privacy-preserving federated learning cyber-threat detection for intelligent transport systems with blockchain-based security. Expert Syst..

[47] Yaga D., Mell P., Roby N., Scarfone K. (2019). Blockchain technology overview. arXiv.

[48] Prajapati P., Shah P. (2022). A review on secure data deduplication: Cloud storage security issue. J. King Saud-Univ.-Comput. Inf. Sci..

[49] Han J., Haihong E., Le G., Du J. Survey on NoSQL database. Proceedings of the 2011 6th International Conference on Pervasive Computing and Applications.

[50] Nayak A., Poriya A., Poojary D. (2013). Type of NOSQL databases and its comparison with relational databases. Int. J. Appl. Inf. Syst..

[51] Mohamed M.A., Altrafi O.G., Ismail M.O. (2014). Relational vs. nosql databases: A survey. Int. J. Comput. Inf. Technol..

[52] Boicea A., Radulescu F., Agapin L.I. MongoDB vs Oracle–database comparison. Proceedings of the 2012 Third International Conference on Emerging Intelligent Data and Web Technologies.

[53] Chowdhury M.J.M., Colman A., Kabir M.A., Han J., Sarda P. Blockchain versus database: A critical analysis. Proceedings of the 2018 17th IEEE International Conference on Trust, Security and Privacy in Computing and Communications/12th IEEE International Conference on Big Data Science and Engineering (TrustCom/BigDataSE).

[54] Namane S., Ahmim M., Kondoro A., Dhaou I.B. (2023). Blockchain-Based Authentication Scheme for Collaborative Traffic Light Systems Using Fog Computing. Electronics.

[55] Singh R., Sturley S., Sharma B., Dhaou I.B. Blockchain-enabled Device Authentication and Authorisation for Internet of Things. Proceedings of the 2023 1st International Conference on Advanced Innovations in Smart Cities (ICAISC).

[56] Ren Y., Liu Y., Ji S., Sangaiah A.K., Wang J. (2018). Incentive mechanism of data storage based on blockchain for wireless sensor networks. Mob. Inf. Syst..

[57] Vokerla R.R., Shanmugam B., Azam S., Karim A., Boer F.D., Jonkman M., Faisal F. An Overview of Blockchain Applications and Attacks. Proceedings of the 2019 International Conference on Vision towards Emerging Trends in Communication and Networking (ViTECoN).

[58] Krichen M. (2023). Strengthening the security of smart contracts through the power of artificial intelligence. Computers.

[59] Krichen M., Lahami M., Al-Haija Q.A. Formal Methods for the Verification of Smart Contracts: A Review. Proceedings of the 2022 15th International Conference on Security of Information and Networks (SIN).

[60] Dolev D. (1982). The Byzantine generals strike again. J. Algorithms.

[61] Monrat A.A., Schelén O., Andersson K. (2019). A Survey of Blockchain From the Perspectives of Applications, Challenges, and Opportunities. IEEE Access.

[62] Bansal P., Panchal R., Bassi S., Kumar A. Blockchain for Cybersecurity: A Comprehensive Survey. Proceedings of the 2020 IEEE 9th International Conference on Communication Systems and Network Technologies (CSNT).

[63] Bains P. (2022). Blockchain Consensus Mechanisms: A Primer for Supervisors.

[64] Wang Q., Huang J., Wang S., Chen Y., Zhang P., He L. (2020). A comparative study of blockchain consensus algorithms. Journal of Physics: Conference Series.

[65] Castro M., Liskov B. Practical byzantine fault tolerance. Proceedings of the OsDI.

[66] Sharma V., Lal N. (2020). A novel comparison of consensus algorithms in blockchain. Adv. Appl. Math. Sci..

[67] Zou W., Lo D., Kochhar P.S., Le X.B.D., Xia X., Feng Y., Chen Z., Xu B. (2019). Smart contract development: Challenges and opportunities. IEEE Trans. Softw. Eng..

[68] Sarmah S.S. (2018). Understanding blockchain technology. Comput. Sci. Eng..

[69] Yalla S.T., Nikhilendra P. (2020). An overview on Blockchain technology and its applications. Proceedings of the ICDSMLA 2019: Proceedings of the 1st International Conference on Data Science, Machine Learning and Applications.

[70] Sheth H., Dattani J. (2019). Overview of blockchain technology. Asian J. Converg. Technol. (AJCT).

[71] Liu M., Wu K., Xu J.J. (2019). How will blockchain technology impact auditing and accounting: Permissionless versus permissioned blockchain. Curr. Issues Audit..

[72] Bhutta M.N.M., Khwaja A.A., Nadeem A., Ahmad H.F., Khan M.K., Hanif M.A., Song H., Alshamari M., Cao Y. (2021). A survey on blockchain technology: Evolution, architecture and security. IEEE Access.

[73] Liu D., Zhang Y., Jia D., Zhang Q., Zhao X., Rong H. (2022). Toward secure distributed data storage with error locating in blockchain enabled edge computing. Comput. Stand. Interfaces.

[74] Nasab S.S.F., Bahrepour D., Tabbakh S.R.K. A Review on Secure Data Storage and Data Sharing Technics in Blockchain-based IoT Healthcare Systems. Proceedings of the 2022 12th International Conference on Computer and Knowledge Engineering (ICCKE).

[75] Vangipuram S.L., Mohanty S.P., Kougianos E., Ray C. (2022). G-DaM: A Distributed Data Storage with Blockchain Framework for Management of Groundwater Quality Data. Sensors.

[76] Wang J., Chen J., Ren Y., Sharma P.K., Alfarraj O., Tolba A. (2022). Data security storage mechanism based on blockchain industrial Internet of Things. Comput. Ind. Eng..

[77] Sharma A., Kaur P. (2023). Tamper-proof multitenant data storage using blockchain. Peer-to-Peer Netw. Appl..

[78] Guo J., Li C., Luo Y. (2022). Blockchain-assisted caching optimization and data storage methods in edge environment. J. Supercomput..

[79] Wu T., Jourjon G., Thilakarathna K., Yeoh P.L. (2023). MapChain-D: A Distributed Blockchain for IIoT Data Storage and Communications. IEEE Trans. Ind. Inform..

[80] Ren Y., Liu X., Sharma P.K., Alfarraj O., Tolba A., Wang S., Wang J. (2023). Data storage mechanism of industrial IoT based on LRC sharding blockchain. Sci. Rep..

[81] Yahaya A.S., Javaid N., Zeadally S., Farooq H. (2022). Blockchain based optimized data storage with secure communication for Internet of Vehicles considering active, passive, and double spending attacks. Veh. Commun..

[82] Anita N., Vijayalakshmi M. Blockchain security attack: A brief survey. Proceedings of the 2019 10th International Conference on Computing, Communication and Networking Technologies (ICCCNT), IIT.

[83] Sayeed S., Marco-Gisbert H. (2020). Proof of adjourn (poaj): A novel approach to mitigate blockchain attacks. Appl. Sci..

[84] Nicolas K., Wang Y., Giakos G.C., Wei B., Shen H. (2020). Blockchain system defensive overview for double-spend and selfish mining attacks: A systematic approach. IEEE Access.

[85] Sayeed S., Marco-Gisbert H. (2019). Assessing blockchain consensus and security mechanisms against the 51% attack. Appl. Sci..

[86] Bhardwaj A., Shah S.B.H., Shankar A., Alazab M., Kumar M., Gadekallu T.R. (2021). Penetration testing framework for smart contract blockchain. Peer-to-Peer Netw. Appl..

[87] Homoliak I., Venugopalan S., Reijsbergen D., Hum Q., Schumi R., Szalachowski P. (2020). The security reference architecture for blockchains: Toward a standardized model for studying vulnerabilities, threats, and defenses. IEEE Commun. Surv. Tutor..

[88] Chen H., Pendleton M., Njilla L., Xu S. (2020). A survey on ethereum systems security: Vulnerabilities, attacks, and defenses. ACM Comput. Surv. (CSUR).

[89] Samsung Blockchain|Apps—The Official Samsung Galaxy Site. https://www.samsung.com/global/galaxy/apps/samsung-blockchain/.

[90] Faridi A., Siddiqui F. (2020). Improving SPV-Based Cryptocurrency Wallet. Proceedings of the Cybernetics, Cognition and Machine Learning Applications: Proceedings of ICCCMLA 2019.

[91] Introduction to Microsoft Entra Verified ID—Microsoft Entra|Microsoft Learn. https://learn.microsoft.com/en-us/azure/active-directory/verifiable-credentials/decentralized-identifier-overview.

[92] IBM Supply Chain Intelligence Suite—Food Trust. https://www.ibm.com/products/supply-chain-intelligence-suite/food-trust.

[93] Main Home—Everledger. https://everledger.io/.

[94] Kshetri N., Voas J. (2018). Blockchain in developing countries. IT Prof..

[95] Frequently Asked Questions—Follow My Vote. https://followmyvote.com/online-voting-platform-faqs/.

[96] Krichen M. (2023). A Survey on Formal Verification and Validation Techniques for Internet of Things. Appl. Sci..

[97] Krichen M., Cheikhrouhou O., Lahami M., Alroobaea R., Jmal Maâlej A. (2018). Towards a model-based testing framework for the security of internet of things for smart city applications. Proceedings of the Smart Societies, Infrastructure, Technologies and Applications: First International Conference, SCITA 2017, Jeddah, Saudi Arabia, 27–29 November 2017.

[98] Krichen M., Lahami M., Cheikhrouhou O., Alroobaea R., Maâlej A.J. (2020). Security testing of internet of things for smart city applications: A formal approach. Smart Infrastructure and Applications.

[99] Introduction|IOTA Wiki. https://wiki.iota.org/get-started/introduction/iota/introduction/.

[100] Whitepaper|Medicalchain. https://medicalchain.com/en/whitepaper/.

[101] The MediLedger Network. https://www.mediledger.com/.

[102] Sarkar A., Maitra T., Neogy S. (2021). Blockchain in healthcare system: Security issues, attacks and challenges. Blockchain Technology: Applications and Challenges.

[103] How Blockchain Technology Affects Mobile Application Development Experience. https://www.linkedin.com/pulse/how-blockchain-technology-affects-mobile-application-development-.

[104] Suankaewmanee K., Hoang D.T., Niyato D., Sawadsitang S., Wang P., Han Z. Performance analysis and application of mobile blockchain. Proceedings of the 2018 International Conference on Computing, Networking and Communications (ICNC).

[105] Pros and Cons of Blockchain in Mobile App Development—Velvetech. https://www.velvetech.com/blog/blockchain-in-mobile/.

[106] Bhattarai A. (2019). Blockchain in Cybersecurity, Pros, and Cons. https://ssrn.com/abstract=3527922.

[107] Pincheira M., Antonini M., Vecchio M. (2022). Integrating the IoT and blockchain technology for the next generation of mining inspection systems. Sensors.

[108] Maftei A.A., Lavric A., Petrariu A.I., Popa V. (2023). Massive Data Storage Solution for IoT Devices Using Blockchain Technologies. Sensors.

[109] Nguyen D.C., Pathirana P.N., Ding M., Seneviratne A. (2019). Blockchain for secure ehrs sharing of mobile cloud based e-health systems. IEEE Access.

[110] Wang J., Chen J., Xiong N., Alfarraj O., Tolba A., Ren Y. (2023). S-BDS: An effective blockchain-based data storage scheme in zero-trust IoT. ACM Trans. Internet Technol..

[111] Xie M., Yu Y., Chen R., Li H., Wei J., Sun Q. (2022). Accountable outsourcing data storage atop blockchain. Comput. Stand. Interfaces.

[112] Mani V., Ghonge M.M., Chaitanya N.K., Pal O., Sharma M., Mohan S., Ahmadian A. (2022). A new blockchain and fog computing model for blood pressure medical sensor data storage. Comput. Electr. Eng..

[113] Panigrahi A., Sahu B., Panigrahi S.S., Khan M.S., Jena A.K. (2021). Application of Blockchain as a solution to the real-world issues in health care system. Blockchain Technology: Applications and Challenges.

[114] Ahmed I., Darda M., Nath S. (2021). Blockchain: A New Safeguard to Cybersecurity. Blockchain Technology: Applications and Challenges.

[115] Stafford T.F., Treiblmaier H. (2020). Characteristics of a blockchain ecosystem for secure and sharable electronic medical records. IEEE Trans. Eng. Manag..

[116] Hakak S., Khan W.Z., Gilkar G.A., Imran M., Guizani N. (2020). Securing smart cities through blockchain technology: Architecture, requirements, and challenges. IEEE Net..

[117] Krichen M. (2010). A formal framework for conformance testing of distributed real-time systems. Principles of Distributed Systems, Proceedings of the International Conference on Principles of Distributed Systems, Tozeur, Tunisia, 14–17 December 2010.

[118] Benisi N.Z., Aminian M., Javadi B. (2020). Blockchain-based decentralized storage networks: A survey. J. Netw. Comput. Appl..

[119] Ye H., Park S. (2021). Reliable vehicle data storage using blockchain and IPFS. Electronics.

[120] Kadëna E. Blockchain integration into mobile devices. Rajnai Zoltán Kiberbiztonság–Cybersecurity 2.

[121] Xu X., Pautasso C., Zhu L., Lu Q., Weber I. A pattern collection for blockchain-based applications. Proceedings of the 23rd European Conference on Pattern Languages of Programs.

[122] Wan Z., Lo D., Xia X., Cai L. Bug characteristics in blockchain systems: A large-scale empirical study. Proceedings of the 2017 IEEE/ACM 14th International Conference on Mining Software Repositories (MSR).

[123] Taş R., Tanrıöver Ö.Ö. (2020). A systematic review of challenges and opportunities of blockchain for E-voting. Symmetry.

[124] Xia P., Wang H., Yu Z., Liu X., Luo X., Xu G. (2021). Ethereum name service: The good, the bad, and the ugly. arXiv.

[125] Sayeed S., Marco-Gisbert H., Caira T. (2020). Smart contract: Attacks and protections. IEEE Access.

[126] Morrison R., Mazey N.C., Wingreen S.C. (2020). The DAO controversy: The case for a new species of corporate governance?. Front. Blockchain.

[127] Scherer M. (2017). Performance and Scalability of Blockchain Networks and Smart Contracts. Ph.D. Thesis.

[128] Kuzlu M., Pipattanasomporn M., Gurses L., Rahman S. Performance analysis of a hyperledger fabric blockchain framework: Throughput, latency and scalability. Proceedings of the 2019 IEEE International Conference on Blockchain (Blockchain).

[129] Yang D., Long C., Xu H., Peng S. A review on scalability of blockchain. Proceedings of the 2020 the 2nd International Conference on Blockchain Technology.

[130] Monrat A.A., Schelén O., Andersson K. Performance evaluation of permissioned blockchain platforms. Proceedings of the 2020 IEEE Asia-Pacific Conference on Computer Science and Data Engineering (CSDE).

[131] Chauhan A., Malviya O.P., Verma M., Mor T.S. Blockchain and scalability. Proceedings of the 2018 IEEE International Conference on Software Quality, Reliability and Security Companion (QRS-C).

[132] Koteska B., Karafiloski E., Mishev A. Blockchain implementation quality challenges: A literature. Proceedings of the SQAMIA 2017: 6th Workshop of Software Quality, Analysis, Monitoring, Improvement, and Applications.

[133] Zhou Q., Huang H., Zheng Z., Bian J. (2020). Solutions to scalability of blockchain: A survey. IEEE Access.

[134] Kohad H., Kumar S., Ambhaikar A. (2020). Scalability issues of blockchain technology. Int. J. Eng. Adv. Technol..

[135] Dabbagh M., Choo K.K.R., Beheshti A., Tahir M., Safa N.S. (2021). A survey of empirical performance evaluation of permissioned blockchain platforms: Challenges and opportunities. Comput. Secur..

[136] Zamani M., Movahedi M., Raykova M. Rapidchain: Scaling blockchain via full sharding. Proceedings of the P2018 ACM SIGSAC Conference on Computer and Communications Security.

[137] Qiu H., Qiu M., Memmi G., Ming Z., Liu M. (2018). A dynamic scalable blockchain based communication architecture for IoT. Proceedings of the Smart Blockchain: First International Conference, SmartBlock 2018.

[138] Chan W.K., Chin J.J., Goh V.T. (2021). Simple and scalable blockchain with privacy. J. Inf. Secur. Appl..

[139] Palm E., Schelén O., Bodin U. Selective blockchain transaction pruning and state derivability. Proceedings of the 2018 Crypto Valley Conference on Blockchain Technology (CVCBT).

[140] Reddy B.S. securePrune: Secure block pruning in UTXO based blockchains using Accumulators. Proceedings of the 2021 International Conference on COMmunication Systems & NETworkS (COMSNETS).

[141] Gruber D., Li W., Karame G. Unifying lightweight blockchain client implementations. Proceedings of the NDSS Workshop Decentralized IoT Security Stand.

[142] Zhao Y., Niu B., Li P., Fan X. (2020). A novel enhanced lightweight node for blockchain. Proceedings of the Blockchain and Trustworthy Systems: First International Conference, BlockSys 2019.

[143] Khan S., Lee W.K., Hwang S.O. (2021). AEchain: A lightweight blockchain for IoT applications. IEEE Consum. Electron. Mag..

[144] Wang Y. (2020). A blockchain system with lightweight full node based on dew computing. IoT.

[145] Na D., Park S. (2021). Fusion chain: A decentralized lightweight blockchain for IoT security and privacy. Electronics.

[146] Liu Y., Wang K., Lin Y., Xu W. (2019). A lightweight blockchain system for industrial internet of things. IEEE Trans. Ind. Inform..

[147] López-Pimentel J.C., Rojas O., Monroy R. Blockchain and off-chain: A solution for audit issues in supply chain systems. Proceedings of the 2020 IEEE International Conference on Blockchain (Blockchain).

[148] Hepp T., Sharinghousen M., Ehret P., Schoenhals A., Gipp B. (2018). On-chain vs. off-chain storage for supply-and blockchain integration. IT-Inf. Technol..

[149] Liu C., Bodorik P., Jutla D. A tool for moving blockchain computations off-chain. Proceedings of the 3rd ACM International Symposium on Blockchain and Secure Critical Infrastructure.

[150] Singh S.K., Jenamani M., Dasgupta D., Das S. (2021). A conceptual model for Indian public distribution system using consortium blockchain with on-chain and off-chain trusted data. Inf. Technol. Dev..

[151] Zhang Y., Zhang L., Liu Y., Luo X. (2021). Proof of service power: A blockchain consensus for cloud manufacturing. J. Manuf. Syst..

[152] Oyinloye D.P., Teh J.S., Jamil N., Alawida M. (2021). Blockchain consensus: An overview of alternative protocols. Symmetry.

[153] Yu L., Zhao X.f., Jin Y., Cai H.y., Wei B., Hu B. (2019). Low powered blockchain consensus protocols based on consistent hash. Front. Inf. Technol. Electron. Eng..

[154] Jiang Y., Ding S. A high performance consensus algorithm for consortium blockchain. Proceedings of the 2018 IEEE 4th International Conference on Computer and Communications (ICCC).

[155] Goel S., Singh A., Garg R., Verma M., Jayachandran P. Resource fairness and prioritization of transactions in permissioned blockchain systems (industry track). Proceedings of the 19th International Middleware Conference Industry.

[156] Khalid R., Javaid N., Almogren A., Javed M.U., Javaid S., Zuair M. (2020). A blockchain-based load balancing in decentralized hybrid P2P energy trading market in smart grid. IEEE Access.

[157] Yahaya A.S., Javaid N., Javed M.U., Shafiq M., Khan W.Z., Aalsalem M.Y. (2020). Blockchain-based energy trading and load balancing using contract theory and reputation in a smart community. IEEE Access.

[158] Inayat K., Hwang S.O. (2018). Load balancing in decentralized smart grid trade system using blockchain. J. Intell. Fuzzy Syst..

[159] Wickham M., Wickham M. (2018). Lazy loading images. Practical Android: 14 Complete Projects on Advanced Techniques and Approaches.

[160] Yu G., Wang X., Yu K., Ni W., Zhang J.A., Liu R.P. (2020). Survey: Sharding in blockchains. IEEE Access.

